# Development of a nucleocapsid protein-based competitive ELISA for the detection of porcine deltacoronavirus antibodies

**DOI:** 10.3389/fmicb.2025.1680835

**Published:** 2025-11-26

**Authors:** Shuizhong Han, Yu Zhou, Maiping Li, Penggang Chen, Xingming Huang, Ying Wang

**Affiliations:** 1 Yan'an Vocational and Technical College, Yan'an, China; 2 College of Veterinary Medicine, Northwest A&F University, Yangling, China; 3 Animal Disease Control Center of Yan'an, Yan'an, China; 4 Animal Husbandry and Veterinary Service Center of Baota District, Yan'an, China

**Keywords:** porcine deltacoronavirus, competitive ELISA, monoclonal antibody, N protein, antibodies

## Abstract

Porcine deltacoronavirus (PDCoV) is a significant threat to the global swine industry, causing severe diarrhea in piglets and exhibiting a broad host range with zoonotic potential. The nucleocapsid (N) protein of PDCoV is highly conserved and immunogenic, making it an ideal target for diagnostic development. However, existing enzyme-linked immunosorbent assays (ELISAs) typically rely on enzyme-labeled secondary antibodies, complicating the experimental procedure. In this study, a monoclonal antibody (mAb 3C6) against the PDCoV N protein was developed and utilized to establish a competitive ELISA (cELISA). The recombinant N protein (~40 kDa) was successfully expressed and purified. Epitope mapping identified the linear B-cell epitope recognized by mAb 3C6 as 121-HQLLPLRFPTGDGPA-135. Using 60 PDCoV-negative serum samples, the cut-off value for the cELISA was determined to be 44.3%. Validation with 175 field swine serum samples demonstrated a diagnostic sensitivity of 88.5%, specificity of 92.6%, and an overall agreement of 92.0%. The cELISA showed analytical sensitivity twice that of an indirect immunofluorescence assay (IFA) and detected PDCoV-specific antibodies as early as 7 days post-infection. No cross-reactivity with other common swine pathogens was observed. The coefficients of variation for intra- and inter-assay reproducibility ranged from 4.39 to 9.20% and 4.20 to 7.97%, respectively. In conclusion, the developed N protein-based cELISA represents a reliable and efficient tool for the serological detection of PDCoV.

## 1 Introduction

Porcine deltacoronavirus (PDCoV), an enveloped, single-stranded, positive-sense RNA virus, belongs to the genus *Deltacoronavirus* within the family *Coronaviridae* (Jung et al., 2016). The prototype PDCoV was initially identified in Hong Kong during a molecular epidemiology study in 2009 (Woo et al., 2012). However, its pathogenic significance was not recognized until 2014 when it was associated with outbreaks of severe diarrhea in swine herds in Ohio, United States (Marthaler et al., 2014b; Wang et al., 2014).

PDCoV infection causes damage to intestinal epithelial cells and leads to clinical signs such as anorexia, vomiting, lethargy, and diarrhea in both gnotobiotic and conventional piglets, whereas growing pigs typically remain asymptomatic (Jung et al., 2015; Dong et al., 2016; Yen et al., 2022). Similar to porcine epidemic diarrhea virus (PEDV) and transmissible gastroenteritis virus (TGEV), PDCoV is mainly transmitted through the fecal - oral route (Alonso et al., 2014; Kikuti et al., 2024). Co-infections involving PDCoV, PEDV, TGEV, and porcine rotavirus (PoRV) are frequently observed in natural swine populations (Ajayi et al., 2018; Zhang et al., 2024), and PDCoV-PEDV co-infection exacerbates the severity of diarrheal in piglets (Zhang et al., 2022). Notably, the clinical signs and histopathological lesions caused by PDCoV are indistinguishable from those induced by PEDV and TGEV (Jung et al., 2015; Wang et al., 2019). Although PDCoV has a lower field prevalence and disease severity compared to PEDV, it has a remarkably broader host range (Kong et al., 2022; Zhang et al., 2024). Besides swine, it can infect chickens, turkeys, and calves (Jung et al., 2017; Liang et al., 2019; Boley et al., 2020). Recently, PDCoV was firstly detected in the plasma samples from Haitian children with acute febrile illness, indicating its zoonotic potential (Lednicky et al., 2021). Currently, PDCoV has been found in many regions, including Canada, South Korea, mainland China, Thailand, Vietnam, Laos, Taiwan, Japan, Mexico, and Haiti, posing a great threat to the global pig industry (Kong et al., 2022).

The PDCoV genome is approximately 25.4 kb in length and encodes four structural proteins, namely, spike (S), envelope (E), membrane (M), and nucleocapsid (N), and two non-structural proteins (NS6 and NS7) (Marthaler et al., 2014a). Among these, the N protein is the most abundant and multifunctional structural component (McBride et al., 2014). It plays a crucial role in viral genome packaging, forming protective ribonucleoprotein complexes that ensure genomic stability, efficient replication, and reliable transmission (Lee and Lee, 2015). Notably, the N protein exhibits strong immunogenicity, inducing detectable serum antibodies as early as 7 days after infection (Zhao et al., 2020). Due to its high conservation across PDCoV strains and potent antigenicity, the N protein is an ideal target for diagnostic development.

The increasing concerns about PDCoV infection have promoted the development of various diagnostic techniques, including virus isolation, cytopathic effect observation, real-time quantitative PCR, enzyme-linked immunosorbent assay (ELISA), immunohistochemistry (Zhang, 2016), which are essential for controlling PDCoV infection. Among serological methods, ELISA is regarded as the most sensitive and specific technique for detecting PDCoV serum antibodies (Zhang, 2016). Several indirect ELISA (iELISA) formats have been developed using different coating antigens, such as whole virus (Ma et al., 2016), S protein (Thachil et al., 2015), M protein (Castaneda Montes et al., 2024), and N protein (Su et al., 2016; Wang et al., 2025a; Wang et al., 2025b). In addition, a blocking ELISA based on a monoclonal antibody against the N protein has been established, though it still requires an anti-mouse conjugate (Wang et al., 2022). Despite the satisfactory performance of existing ELISAs, their reliance on enzyme-labeled secondary antibodies complicates the assay procedure and prolongs the detection time.

To address this limitation, the present study developed a monoclonal antibody (mAb) against the PDCoV N protein and conjugated it with horseradish peroxidase (HRP). The HRP-mAb complex was subsequently employed to establish a competitive ELISA (cELISA) for PDCoV serological detection.

## 2 Materials and methods

### 2.1 Viruses, cells, mice, and sera

PDCoV strain HN and LLC-PK cells were maintained in our laboratory. LLC-PK cells were grown in Modified Eagle’s medium (MEM) (Gibco, Beijing, China) supplemented with 10% fetal bovine serum (FBS, Hyclone, United States), antibiotics (100 U/mL of penicillin and 100 μg/mL of streptomycin), and trypsin (5 μg/mL) at 37 °C in 5% CO_2_. Mouse myeloma cells SP2/0, also maintained in our laboratory, were cultured in RPMI1640 medium (Gibco, Beijing, China) containing 17% FBS under the same conditions. Eight-week-old female BALB/c mice were purchased from Vital River Laboratory Animal Technology Co., Ltd. (Beijing, China). A total of 60 serum samples were collected from colostrum-deprived piglets confirmed to be PDCoV nucleotide-negative by RT-PCR. Rectal swab samples were analyzed using primers targeting the PDCoV N gene (F: 5′-CCAAACGC AACCCCAACAATCC-3′; R: 5′-CTTCTCAGTGTCTGCAGAGCCG-3′) according to the protocol described previously (Zhang et al., 2024). A total of 175 serum samples used for the cELISA validation were obtained from suckling piglets (7–21 days old) on diarrhea and non-affected pig farms in Shaanxi province between September and December 2024. Swine antisera against PEDV (*n* = 3, neutralizing antibody [NAb] titer 1:16), TGEV (*n* = 3, NAb titer 1:32), PoRV (*n* = 3, NAb titer of 1∶32), African swine fever virus (ASFV) (*n* = 3, S/N ratios: 1.128, 1.352, 1.165), classical swine fever virus (CSFV) (*n* = 3, PI values: 76, 84, 82%), porcine reproductive and respiratory syndrome virus (PRRSV) (*n* = 3, S/*p* values: 0.685, 0.732, and 0.775), porcine pseudorabies virus (PRV) (*n* = 3, NAb titer 1:32) and porcine circovirus 2 (PCV2) (*n* = 3, IFA titer 1:160) were also preserved in our laboratory.

### 2.2 Construction of recombinant pET-28a (+)-PDCoV-N plasmid

The full-length N gene of PDCoV strain HN was amplified and subsequently cloned into the prokaryotic expression pET-28a (+). Briefly, viral RNAs were extracted from PDCoV-infected cell cultures using the TIANamp Virus DNA/RNA Kit (Tiangen Biotech, Beijing, China) according to the manufacturer’s instructions, and then the first-strand cDNA was synthesized with the FastKing RT Kit (with gDNase) (Tiangen Biotech, Beijing, China). Primers were designed based on the PDCoV reference strain (GenBank MN025260) and synthesized by Tsingke Biotechnology Co., Ltd. (Beijing, China): F: 5′-CGCGGATCCATGGCTGCACCAGTAGT-3′; R: 5′-CCGCTCGAGCTACGCTGCTGATTCCT-3′. PCR was performed in a 50 μL reaction mixture containing 2 μL of each primer, 25 μL of 2 × PrimeSTAR Max Premix, 19 μL of ultrapure water, and 2 μL of template DNA. The PCR reaction conditions were as follows: 94 °C for 10 min; 35 cycles of 95 °C for 30 s, 58 °C for 30 s, 72 °C for 60 s, and 72 °C for 10 min. The amplified product was ligated into pET-28a (+) and transformed into *E. coli* DH5α by heat shock method. After sequencing, the pET-28a (+)-PDCoV-N plasmid was transformed into BL21 (DE3) competent cells.

### 2.3 Expression and purification of His-tagged PDCoV N protein

*E. coli* BL21 (DE3) containing pET-28a (+)-PDCoV-N were cultured in LB broth supplemented with kanamycin (50 μg/mL) at 37 °C with shaking at 200 rpm. After induction with 1 mM isopropyl β-D − 1-thiogalactopyranoside (IPTG) at 22 °C for 12 h, cells were harvested by centrifugation at 8000 rpm for 10 min at 4 °C. Cells were washed twice with Tris–HCl (0.02 mol/L, pH 8.0) and sonicated on ice with 5-s pulses at 15-s intervals. After centrifugation, the supernatant was applied to an affinity chromatography column prepacked with Ni-NTA His-Bind® Resin (Huiyan bio, Wuhan, China), and the recombinant protein was eluted with a linear gradient from 20 to 500 mM imidazole. Expression and purification were analyzed by 12% SDS-PAGE, and protein concentration was determined using a BCA Protein Assay kit (Beyotime, China).

### 2.4 Western blotting

The reactivity of the recombinant PDCoV N protein with anti- His mAb and swine anti- PDCoV positive serum was determined using a western blotting assay. Briefly, the recombinant PDCoV N protein was separated by 12% SDS-PAGE and transferred onto polyvinylidene fluoride (PVDF) membranes (Millipore, Billerica, MA, United States). After blocking with 5% (w/v) skimmed milk in TBST (TBS containing 0.1% Tween-20), the membrane was incubated with anti- His mAb (1:500) or swine anti- PDCoV serum (1:200), followed by horseradish peroxidase (HRP)-conjugated goat anti-mouse IgG or rabbit anti-pig IgG (Boster, Hubei, China) at a dilution of 1:10000 for 1 h at room temperature (RT). The binding band was visualized with enhanced chemiluminescence (ECL) using a protein blotting imaging system (Amersham ImageQuant 800, Cytiva).

### 2.5 Preparation of monoclonal antibodies against the PDCoV N protein

The monoclonal antibodies (mAbs) against the PDCoV N protein were generated as previously described (Chang et al., 2025). Briefly, three female BALB/c mice were subcutaneously injected with inactivated PDCoV (10^5.0^ TCID_50_) emulsified with Freund’s complete adjuvant (Sigma, United States) at an equal ratio (w/w) at multiple points on the back. Three booster immunizations with the same dose of antigen plus Freund’s incomplete adjuvant were conducted at two-week intervals. Blood samples were collected from the retro-orbital sinus 2 weeks after the final booster immunization, and the antibody titers were measured by indirect ELISA. When the antibody titers exceeded 1:128000, splenocytes were isolated and fused with SP2/0 myeloma cells using PEG1500.

The fused cells were seeded in 96-well plates and maintained in RPMI1640 medium supplemented with hypoxanthine-aminopterin-thymidine (HAT) and 17% FBS. After 10 days, HAT medium was replaced with HT medium. The hybridoma supernatants were screened were screened by iELISA against PDCoV N protein. Positive hybridoma clones were subcloned by limited dilution method at least three times. The binding capability of the mAb to PDCoV was assessed by Western blotting, indirect immunofluorescence assay (IFA) and immunoperoxidase monolayer assay (IPMA). Cross-reactivity of the mAb with PEDV, TGEV, PoRV, and *E. coli* was evaluated by iELISA.

### 2.6 Enzyme-linked immunosorbent assay

Polystyrene microtiter plates were coated with 1 μg/mL PDCoV N protein diluted in sodium carbonate buffer (0.1 mol/L, pH 9.6) and incubated overnight at 4 °C. After washing three times with PBS supplemented with 0.05% Tween-20 (PBST), the plates were blocked with 5% skim milk in PBST for 2 h at 37 °C. After three washes, the diluted serum samples or hybridoma cell supernatants were added and incubated at 37 °C for 2 h. After three more washes, 1:5000 dilution of HRP-conjugated goat anti-mouse IgG (Boster, Hubei, China) was added and incubated at 37 °C for 1 h. Finally, 3,3′,5,5′-tetramethylbenzidine substrate solution (Beyotime, China) was added, and colorimetric reaction was developed for 15 min at 37 °C. Reaction was terminated by adding 2 mol/L H_2_SO_4_. Finally, the optical density (OD) values were measured at 450 nm.

### 2.7 Indirect immunofluorescence assay

LLC-PK cells were infected with PDCoV strain HN and cultured in a 96-well cell culture dish in MEM medium. After 80% of cells showed typical cytopathic effects (CPEs), the cells were fixed with 80% cold acetone. Then, the cells were blocked with 5% bovine serum albumin (BSA) at RT for 2 h. After washing, mAbs (1:40 dilution) or swine sera (1:20 dilution) were added and incubated at 37 °C for 1 h. The unbound antibodies were discarded, and the plates were washed three times with PBS. Fluorescein isothiocyanate (FITC)–labeled goat anti-mouse IgG or Rabbit anti-Pig IgG (Boster, Hubei, China) was added at a dilution of 1:500 and incubated at 37 °C for 1 h. Finally, the immunofluorescence was detected using an inverted fluorescence microscope (Olympus, Japan).

### 2.8 Immunoperoxidase monolayer assay

IPMA was performed as described previously with some modifications (Han et al., 2016). Briefly, the PDCoV-infected cells were placed on 36-well glass slides and fixed with 80% cold acetone for 10 min at 4 °C. After washing with PBS, mAb (1:40) was added and incubated at 37 °C for 1 h. After washing, HRP-conjugated goat anti-mouse antibody (1:1000) (Boster, Hubei, China) was added and incubated at 37 °C for 0.5 h. After washing, the slides were incubated in diaminobenzidine substrate solution for 5 min at room temperature. Then, the cells were stained with hematoxylin and eosin stain and examined using an inverted light microscope (Olympus, Japan).

### 2.9 Characterization of mAb 3C6 against the PDCoV N protein

Hybridoma supernatants were analyzed for antibody subclass using the Mouse Monoclonal Antibody Subtyping Kit (Proteintech, Wuhan, China). Karyotype analysis was performed on the hybridoma cells using the colchicine inhibition method. Ascites were prepared by injecting hybridoma cells into paraffin-sensitized mice and collected after 7–10 days. The mAb 3C6 titer was determined by iELISA and purified from the ascites using protein G chromatography (GE Healthcare, United States) according to the manufacturer’s instructions.

### 2.10 Identification of B cell epitope recognized by mAb 3C6

To identify the epitope recognized by mAb 3C6, the PDCoV N protein was truncated into a series of fragments. Genes encoding these fragments were synthesized and subsequently inserted into the pGEX-4 T-1 vector by Tsingke Biotechnology Co., Ltd. (Beijing, China). The recombinant GST-tagged proteins were expressed in *E. coli* BL21 (DE3), and their reactivity with mAb 3C6 was detected via Western blotting. To assess the conservation of the mAb 3C6 epitope on the PDCoV N protein, a sequence alignment was performed using DNAstar MegAlign (Madison, WI, United States). The N protein sequences of eight PDCoV strains, isolated from different countries or distinct regions within China, were retrieved from GenBank.

### 2.11 Establishment of competitive ELISA using HRP-conjugated mAb 3C6 against PDCoV N protein

The purified mAb 3C6 was conjugated to HRP using an HRP-labeling kit (Proteintech, Wuhan, China) according to the manufacturer’s instructions. Checkerboard titration was used to optimize the coating concentration of PDCoV N protein and the dilution of HRP-conjugated mAb 3C6. Microtiter plates were coated with 100 μL/well of two-fold serially diluted PDCoV N protein (10–0.3125 μg/mL) and incubated overnight at 4 °C. After three times washing with PBST, plates were blocked with 2% BSA in PBST for 2 h at 37 °C. Following three times washing, 100 μL/well of a mixture containing two-fold serially diluted HRP-conjugated mAb 3C6 (1:500–1:4000) and an equal volume of undiluted PDCoV positive or negative swine serum was added to odd and even columns, respectively, and incubated at 37 °C for 0.5 h. After three times washing, TMB substrate was added, and the colorimetric reaction was developed for 15 min at 37 °C. The reaction was stopped with 2 mol/L H_2_SO_4_, and OD_450nm_ values were measured. Optimal conditions were selected based on the highest negative/positive (N/P) OD ratio and a positive serum OD_450nm_ values approximately 1.0.

To determine the optimal dilution of tested sera, PDCoV positive and negative reference swine sera were serially diluted two-fold (1:5–1:160) and tested as described above. The optimal dilution was selected based on the highest N/P ratio.

### 2.12 Determination of the cut-off value for cELISA

The cut-off value was established using 60 serum samples collected from colostrum-deprived piglets. Percent inhibition (PI) of each sample was calculated using the following formula: PI(%) = [(mean OD_450nm_ of negative control - OD_450nm_ of sample) / (mean OD_450nm_ of negative control]) × 100 (%). The cut-off value was defined as the mean PI value of the 60 negative serum samples plus three standard deviations (SDs), ensuring 99% confidence for negativity.

### 2.13 Sensitivity, specificity and reproducibility of the cELISA

To determine the lowest detection limitation, three PDCoV positive serum samples (IFA titer 1:320) were two-fold serially diluted from 1:10 to 1:2560, and then detected by the established cELISA. The maximum dilution of the serum sample that produced S/N value greater than the cut-off value was defined as the sensitivity of the cELISA. Cross-reactivity was evaluated using antisera against ASFV, CSFV, PRRSV, PRV, PCV2, PEDV, TGEV, and PoRV.

Reproducibility was evaluated by testing 15 serum samples (5 strongly positive, 5 weakly positive, 5 negative). Intra-assay variation was tested in triplicate on one plate; inter-assay variation was tested across three independent runs. Coefficient of variation (CV) were calculated for both.

### 2.14 Detection of antibody response in piglets infected with PDCoV by cELISA

To address the stage of infection detectable by the established cELISA, serum samples from a previous infection study were tested. These serum samples were collected from piglets (21 days old) orally inoculated with 3 mL of PDCoV cultures (10^5.0^ TCID_50_/mL) at 0, 7, 14, and 21 days post infection (dpi).

### 2.15 Validation of the cELISA

A total of 175 field serum samples from diarrheic swine farms in Shaanxi Province were evaluated in parallel by the cELISA and an IFA. The IFA results were independently assessed by two blinded operators, with any discordant samples adjudicated by a third reader. Inter-rater agreement between the initial two operators was analyzed using IBM SPSS Statistics 20 (Armonk, NY, United States). The diagnostic sensitivity, specificity, and overall agreement of the cELISA were calculated against the resolved IFA results.

## 3 Results

### 3.1 Expression, purification, and identification of PDCoV N protein

**Figure 1:**
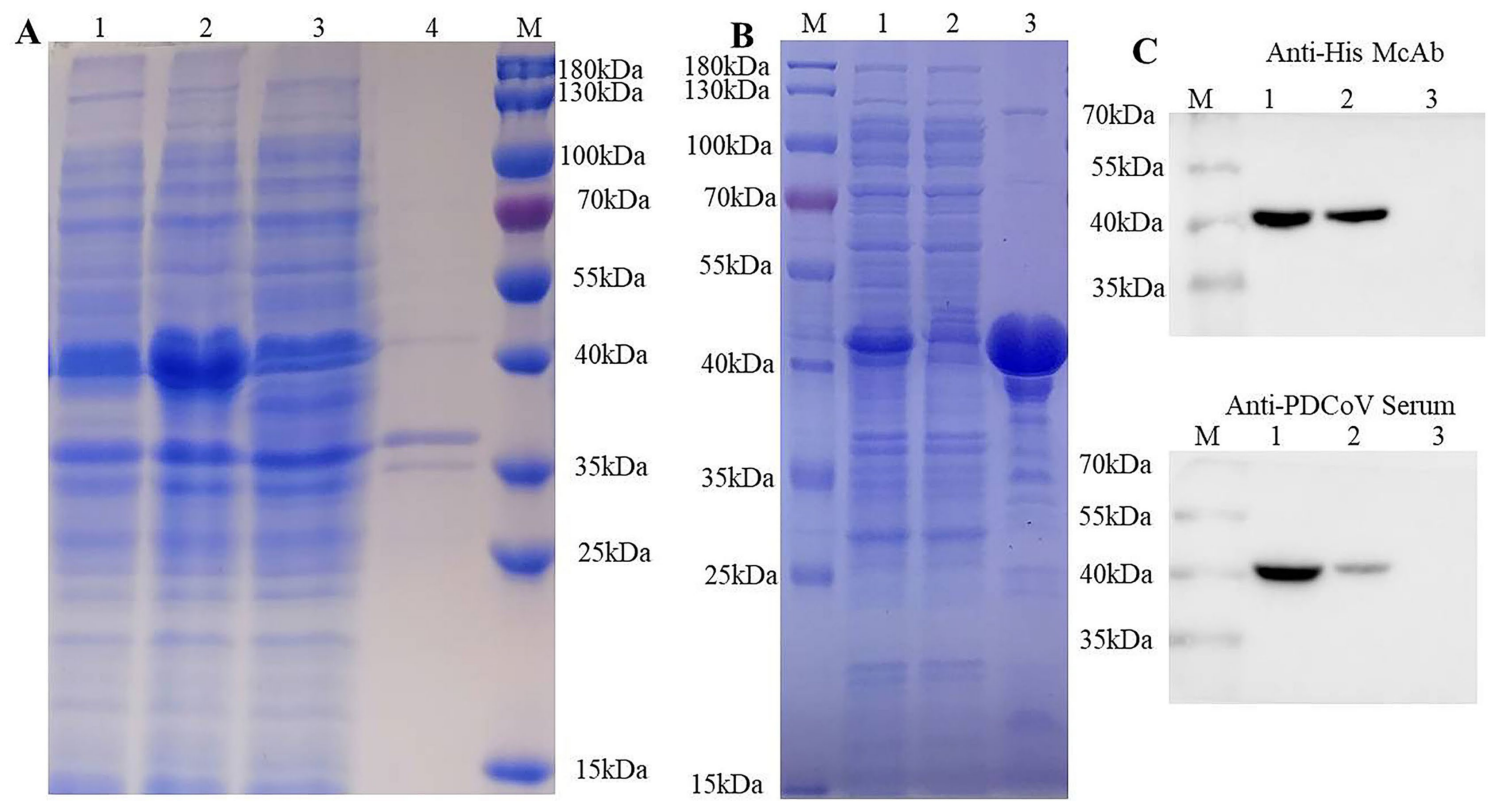
Purification and identification of recombinant PDCoV N protein. **(A)** Solubility analysis of recombinant PDCoV N protein expression in *E. coli*. Lane 1, the whole cell lysates of *E. coli* BL21 (pET-28a(+)-PDCoV-N) before IPTG induction; Lane 2, the whole cell lysates of *E. coli* BL21 (pET-28a(+)-PDCoV-N) after IPTG induction; Lane 3, supernatants from IPTG-induced *E. coli* BL21 (pET-28a(+)-PDCoV-N); Lane 4, sediments from IPTG-induced *E. coli* BL21 (pET-28a(+)-PDCoV-N); Lane M, protein molecular weight marker. **(B)** SDS-PAGE analysis of the purification of PDCoV N protein by Ni^+^ column. Lane M: Protein molecular weight marker; Lane 1, the supernatant of the whole bacterial lysate; Lane 2, elution with 50 mM of imidazole; Lane 3, elution with 500 mM of imidazole. **(C)** Western blotting analysis of the purified PDCoV N protein using anti-His-tag monoclonal antibody or pig anti- PDCoV polyclonal antibodies.

Following induction, the recombinant PDCoV N protein was successfully expressed in *E. coli*, showing a predominant band of approximately 40 kDa in the soluble fraction of the bacterial lysate (Figure 1A). The His-tagged protein was purified using Ni-NTA His-Bind® Resin column and a marked protein band with a molecular weight of about 40 kDa was observed in the eluted fractions (Figure 1B). Western blotting analysis confirmed the specific reactivity of the purified protein with both anti-His mAb and swine anti-PDCoV polyclonal antibodies (Figure 1C). After dialysis, the concentration of the purified recombinant N protein was determined to be 2.3 mg/mL.

### 3.2 Preparation of mAbs against the PDCoV N protein

**Figure 2:**
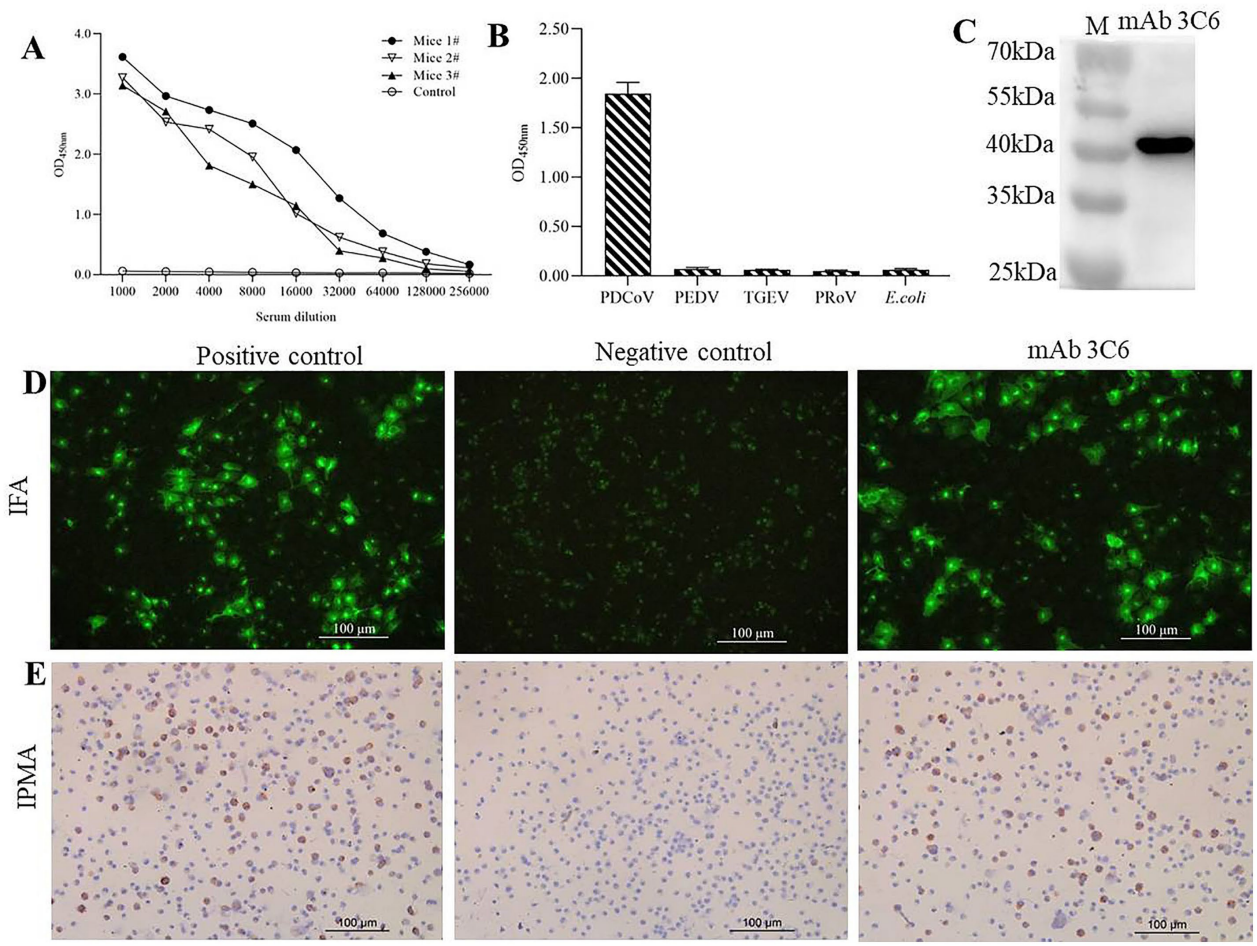
Preparation of mAb 3C6. **(A)** Determination of serum antibody titers in mice by iELISA after the last immunization. **(B)** Reactivity of mAb 3C6 with PDCoV, PEDV, TGEV, PoRV, and *E. coli* by iELISA. **(C)** Reactivity of mAb 3C6 with PDCoV N protein by Western blotting; **(D)** Reactivity of LLC-PK cells infected with the PDCoV strain HN with mAb 3C6 by IFA; **(E)** Reactivity of LLC-PK cells infected with the PDCoV strain HN with mAb 3C6 by IPMA.

Two weeks after the final immunization, serum antibody titers against PDCoV N protein in all immunized mice reached 1:128000, as determined by iELISA (Figure 2A). After three rounds of subcloning, a stable hybridoma clone, designated 3C6, was established. This clone secreted monoclonal antibodies that reacted specifically with PDCoV but showed no cross-reactivity with PEDV, TGEV, PoRV, or *E. coli* antigens in iELISA (Figure 2B). The specificity of mAb 3C6 was further confirmed by Western blotting, which demonstrated its binding to the PDCoV N protein (Figure 2C). Additionally, both IFA and IPMA confirmed the reactivity of mAb 3C6 with PDCoV-infected LLC-PK cells (Figures 2D,E).

### 3.3 Characterization of mAb 3C6

**Figure 3:**
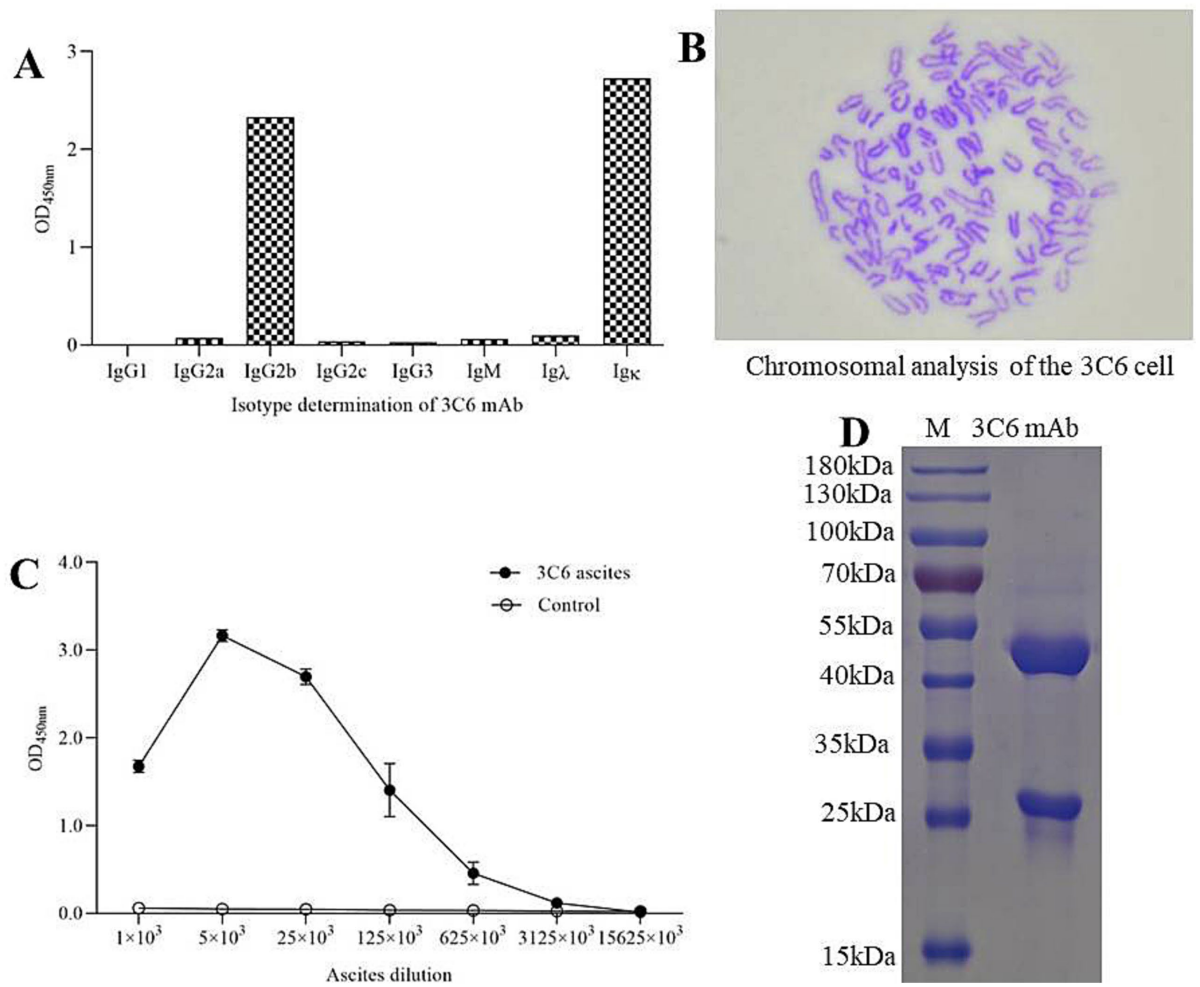
Identification of mAb 3C6. **(A)** Subtype classification of mAb 3C6; **(B)** Chromosomal analysis of the 3C6 cell; **(C)** Determination of ascites antibody titers by iELISA; **(D)** SDS-PAGE analysis of the purity of mAb 3C6 after affinity purification.

Using a commercial subtyping kit, mAb 3C6 was identified as an IgG2b isotype with a kappa light chain (Figure 3A). Karyotype analysis showed that the hybridoma cells contained 96–105 chromosomes (Figure 3B). The titer of ascites produced by the 3C6 hybridoma cells, as determined by iELISA, exceeded 3.125 × 10^6^ (Figure 3C). SDS-PAGE analysis of the protein G-purified antibody from ascites revealed clear bands at approximately 50 kDa and 25 kDa, corresponding to the heavy and light chains of IgG, respectively (Figure 3D).

### 3.4 Epitope mapping of mAb 3C6 against the PDCoV N protein

**Figure 4:**
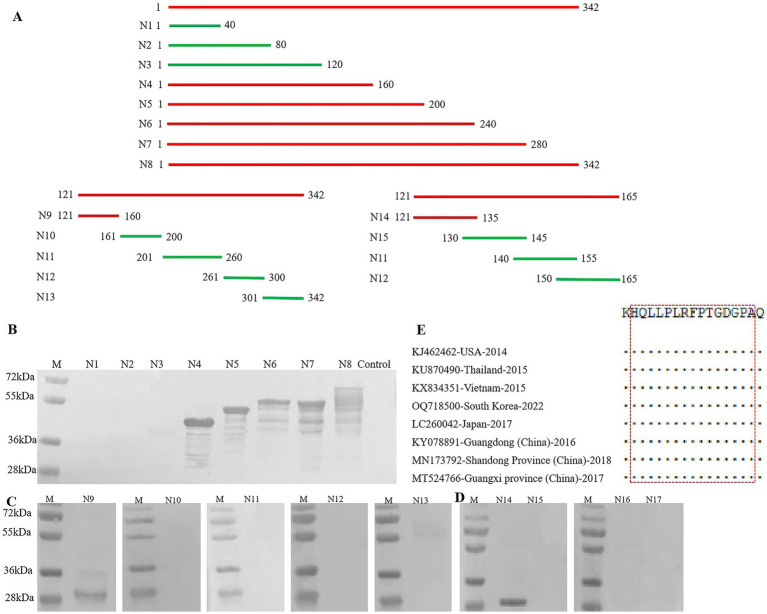
Peptide mapping of the linear epitope recognized by mAb 3C6 against PDCoV N protein. **(A)** Schematic diagram of the relative locations of truncated fragments of PDCoV N protein for epitope mapping. The segments that could be recognized by mAb 3C6 are highlighted in red; and the segments in green could not react with mAb 3C6. **(B–D)** The segments of PDCoV N protein (N1-N17) were analyzed by Western blotting. **(E)** Conservation analysis of the mAb 3C6 epitope in different PDCoV strains.

Three rounds of Western blotting were performed using the expressed truncated PDCoV N protein fragments (Figure 4A). The GST-fused fragments were successfully expressed in *E. coli* (Supplementary Figures S1–S3). In the first round, Western blotting results showed that mAb 3C6 did not recognize fragments N1–N3, indicating that the epitope for mAb 3C6 was localized to the region spanning amino acids (aa) 121–342 (Figure 4B). To narrow down the epitope, the aa 121–342 region was further truncated into five smaller fragments (designated N9–N13) in the second round. Western blotting revealed that the GST-tagged fragment corresponding to aa 121–165 could react with mAb 3C6 (Figure 4C). For further refinement of the epitope, the aa 121–165 region was truncated into four overlapping peptides. The peptide corresponding to 121-HQLLPLRFPTGDGPA-135 was recognized by mAb 3C6 (Figure 4D). Multiple alignments revealed that the epitope is highly conserved across all analyzed PDCoV strains, and shared 100% sequence similarity (Figure 4E).

### 3.5 Establishment of a competitive ELISA using HRP-labeled mAb 3C6 against PDCoV N protein

**Figure 5:**
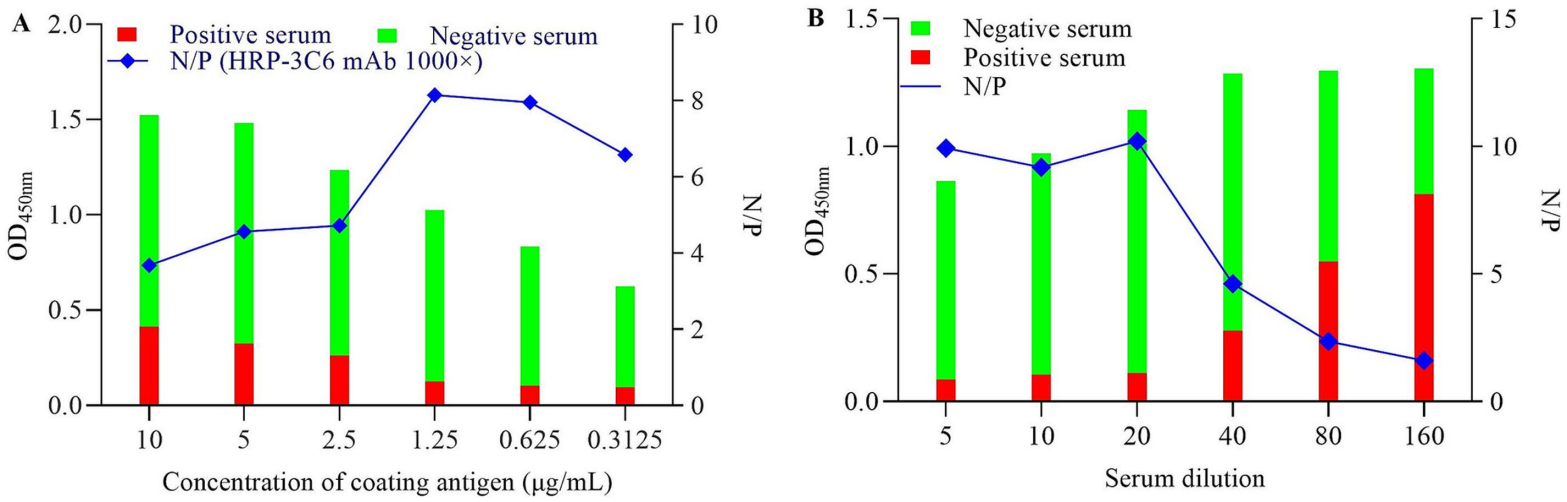
Optimization of the cELISA using HRP- conjugated mAb 3C6 against PDCoV N protein. **(A)** Determination of the optimal coating concentration of PDCoV N protein and the dilution of HRP-conjugated mAb 3C6; **(B)** Determination of the optimal dilution of the tested sera.

Checkerboard titration assay was used to determine the optimal coating concentration of PDCoV N protein, the dilution of HRP-conjugated mAb 3C6, and the dilution of tested sera. The results showed that PDCoV N protein coated at 1.25 μg/mL and 1:1000 dilutions of HRP-conjugated mAb 3C6 yielded the highest N/P ratio (8.143), with OD_450nm_ values of 1.026 (negative serum) and 0.126 (positive serum) (Figure 5A). Moreover, a 1:20 serum dilution was optimal, with a high N/P ratio (10.205), an OD_450nm_ of 1.143 (negative serum) and an OD_450nm_ of 0.112 (positive serum) (Figure 5B).

### 3.6 Determination of the cut-off value for the cELISA

The 60 PDCoV-negative serum samples were tested by cELISA. Analysis revealed that a PI value of 38.2% (mean + 2SD: 26.1% + 12.1%) provided 95% confidence (58/60 negative), whereas a PI of 44.3% (mean + 3SD) achieved 99% confidence (60/60 negative).

### 3.7 Sensitivity, specificity, and reproducibility of the cELISA

**Figure 6:**
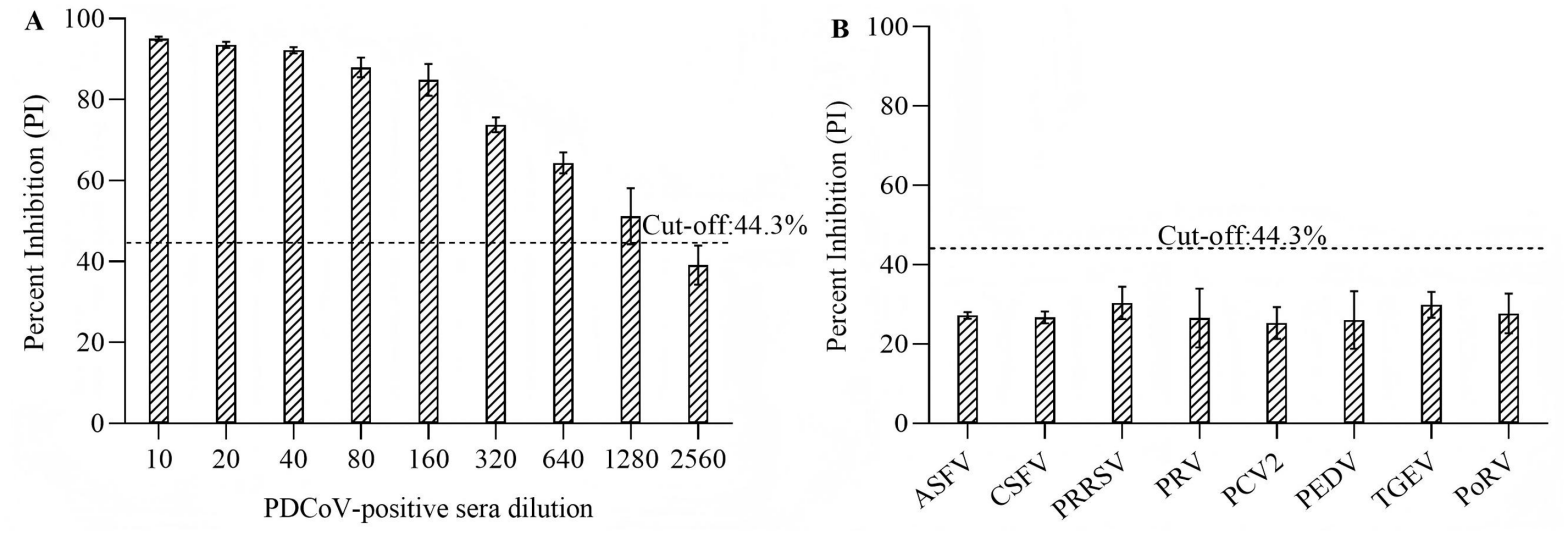
Sensitivity and specificity of the cELISA. **(A)** Two-fold serial dilutions of three PDCoV-positive serum samples (IFA titers: 1:320) were tested by cELISA; **(B)** Antisera against ASFV, CSFV, PRRSV, PRV, PCV2, PEDV, TGEV, and PoRV were tested by cELISA.

To evaluate the sensitivity of the developed cELISA, two-fold serial dilutions of three PDCoV-positive serum samples with IFA titers 1:320 were tested by the developed cELISA. The positive reactions were observed at the dilution of 1:640 or 1:1280 in cELISA (Figure 6A). To confirm the specificity, the cELISA was used to detect sera against ASFV, CSFV, PRRSV, PRV, PCV2, PEDV, TGEV, and PoRV sera, and no cross-reactivity was observed (Figure 6B). The reproducibility of cELISA was determined by calculating the CV of the 15 serum samples PI values. The intra-assay CVs ranged from 4.39 to 9.20%, and inter-assay CVs ranged from 4.20 to 7.97% (Table 1).

**Table 1:** Coefficient values of the 15 serum samples tested by the cELISA.

Sera	No. of sera tested	CV rang (%)	
		Inter-assay	Intra-assay
Strongly positive	5	4.20–7.70	5.35–8.52
Weakly positive	5	5.70–7.97	4.50–9.20
Negative	5	3.74–6.02	4.39–7.15

### 3.8 Detection of antibody response in piglets infected with PDCoV by cELISA

**Figure 7:**
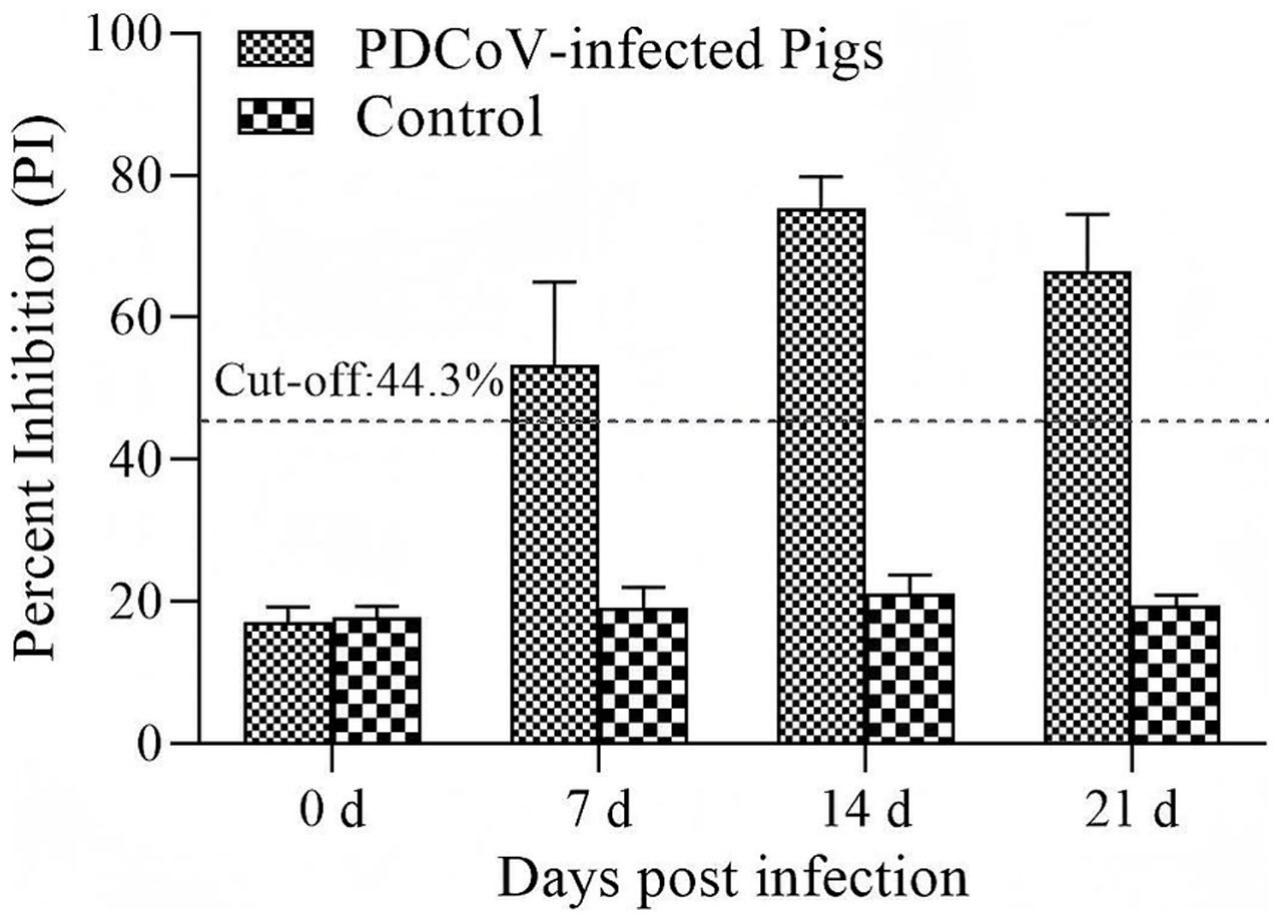
Detection of antibody response against PDCoV infection. The dashed line represents the cut-off of cELISA (PI ≥ 44.3% is positive, otherwise, is negative). Bars represent the mean ± standard deviations of the PI values of samples in each group.

Serum samples collected from piglets orally inoculated with PDCoV strain HN were tested by cELISA. The piglets seroconverted (3/5) at 7 dpi, peaked (5/5) at 14 dpi, and decreased at day 21 (5/5) (Figure 7).

### 3.9 Validation of the cELISA

To investigate PDCoV seroprevalence, 175 field serum samples from diarrheic swine farms were analyzed by cELISA and IFA. The reliability of the IFA was confirmed by an almost perfect inter-operator agreement (Cohen’s kappa = 0.889, *p* < 0.05) (Supplementary Table S1). The final IFA results indicated a positive rate of 14.9% (26/175), and the cELISA yielded a positive rate of 19.4% (34/175). When compared against this IFA reference, the cELISA demonstrated a sensitivity of 88.5% (23/26), a specificity of 92.6% (138/149), and an overall agreement of 92.0% (161/175) (Table 2).

**Table 2:** Prevalence of PDCoV infection in diarrhea-affected swine farms.

cELISA	IFA		Sensitivity (%)	Specificity (%)	Accuracy (%)
	Positive	Negative			
Positive	23	11	88.5% (23/26)		
Negative	3	138		92.6% (138/149)	
Total	26	149			92.0% (161/175)

## 4 Discussion

Since the initial report of PDCoV-associated diarrhea in swine in 2014, the virus rapidly spread to multiple states across the United States (Ma et al., 2015). To date, PDCoV has been found widely circulating in pigs in the North America, East Asia and Southeast Asia (Kong et al., 2022). With a 40–60% mortality rate in suckling piglets (Jung et al., 2015; Ma et al., 2015), PDCoV poses a severe threat to the global pig industry. In mainland China, PDCoV was first reported in 2015 (Dong et al., 2015). Subsequent surveillance data detected a 17.14% (307/1791) positive rate in diarrheal samples from southern China between 2021 and 2023 (Zhang et al., 2024). Given the frequent co-infections and overlapping epidemiological, clinical, and pathological features of PDCoV, PEDV, and TGEV, laboratory diagnosis of PDCoV primarily relies on virological and serological assays (Jung et al., 2016; Zhang et al., 2024). ELISA is widely used in epidemiological investigations, infection monitoring, and immune status evaluation. To date, several types of iELISAs and a blocking ELISA have been developed for antibody detection (Thachil et al., 2015; Ma et al., 2016; Su et al., 2016; Wang et al., 2022; Castaneda Montes et al., 2024; Wang et al., 2025a; Wang et al., 2025b). Nevertheless, the antigenic cross - reactivity between PDCoV and PEDV has the potential to reduce the accuracy of iELISA (Ma et al., 2016; Wang et al., 2025b). Furthermore, the requirement for enzyme-labeled secondary antibodies complicates the assay procedure and increases the overall detection time. In the present study, a cELISA using HRP-labeled mAb against the PDCoV N was established, and can effectively avoid these limitations.

The PDCoV N protein is highly conserved, strongly immunogenic, and a major structural component of the virion (Sun et al., 2020; Wang et al., 2022), making it a promising diagnostic antigen. However, a certain degree of serological cross-reactivity between PDCoV and PEDV has been reported, which can compromise diagnostic specificity when using the whole virus as an antigen (Ma et al., 2016; Wang et al., 2025a; Wang et al., 2025b). Studies have identified that four epitopes on the N protein contribute to this cross-reactivity (Ma et al., 2016; Wang et al., 2025b). It was reported that the ELISA cross-reactivity rate was 27.59% in the detection of PDCoV positive sera with clear background using the full-length N protein (Wang et al., 2025b). However, this cross-reactivity was significantly reduced when using a truncated segment (aa 166–342) and was notably absent with a shorter fragment (aa 217–342) (Wang et al., 2025a; Wang et al., 2025b). In contrast, another study observed no cross-reactivity with the full-length protein (Su et al., 2016), a discrepancy potentially attributable to differences in the sera background. Compared with the truncated protein, ELISA based on the full-length protein would exhibit higher sensitivity in the detection of PDCoV antibodies. Additionally, the use of mAb can significantly mitigate cross-reactivity by targeting a single, specific epitope. Therefore, the full-length PDCoV N protein was used in conjunction with a specific mAb to establish a cELISA in this study.

The selection of antigens is crucial in the preparation of mAbs. In the present study, the PDCoV strain HN was utilized to generate specific monoclonal antibodies in mice. Compared with the recombinant PDCoV N protein, PDCoV retains the natural conformation, which would induce antibodies resembling those produced during natural infection. Hybridomas were screened by iELISA based on the PDCoV N protein and a serious of mAbs was obtained. The results showed that mAb 3C6 specifically recognized PDCoV N protein without cross-reactivity to PEDV, TGEV, or PoRV. To identify the epitope recognized by mAb 3C6, the N protein was systematically truncated into three groups of fragments and expressed in *E. coli*. Through three rounds of Western blotting analysis, the epitope was identified as the linear sequence 121-HQLLPLRFPTGDGPA-135. Several B-cell epitopes on the PDCoV N protein have been previously reported, including 28-QFRGNGVPLNSAIKPVE-44, 59-GTPIPPSYAFYY-70, 251-NFQAG-PDYER-276, 309-NKRETTLQQ-317, and 326-QDWEWDDA-333 (Fu et al., 2020; Wei et al., 2021; Ren et al., 2022; He et al., 2023). Furthermore, multiple sequence alignment revealed that this epitope is highly conserved across PDCoV strains, underscoring the potential of mAb 3C6 as a universal reagent for PDCoV detection. The structural and functional implications of this epitope warrant further investigation.

The present ELISAs for the detection of PDCoV antibodies often require the pre-incubation of the tested sera with the coating antigen, then, the binding antibodies were detected by enzyme-labeled secondary antibodies targeting the species. In cELISA, the secondary antibody against the tested species is nonessential, and only 0.5 h is required prior to colorimetric reaction development, whereas over 3 h is needed for this step in blocking ELISA and 1–2 h in iELISA (Thachil et al., 2015; Ma et al., 2016; Su et al., 2016; Wang et al., 2022; Castaneda Montes et al., 2024). The direct HRP conjugation in the cELISA significantly reduced the time-to-result. To evaluate the stage of infection detectable by the cELISA, serum samples from piglets experimentally infected with PDCoV were detected. The cELISA can detect PDCoV infection as early as 7 dpi. This result differs from a previous report in which IgG antibodies were detected by iELISA using the whole PDCoV at 14 dpi, despite neutralizing antibodies seroconverting by 7 dpi (Hu et al., 2016). This discrepancy may be attributed to differences in the challenge strain, piglet age, or the antigen used in the ELISA. Notably, PDCoV nucleic acid was detected by RT-PCR in fecal samples from all infected piglets (5/5) at 7 dpi (data not shown), whereas the cELISA showed a 60% positive rate (3/5) at the same time point. This observation suggests that RT-PCR offers higher sensitivity for direct viral detection, while the cELISA serves as a valuable tool for monitoring specific serological responses during infection.

In the validation of cELISA, 175 field serum samples where all samples, including discordant ones, were included. The five samples with initially discordant IFA readings were re-evaluated and definitively classified by a third, independent reader. When the cELISA results were compared against this resolved reference standard, the performance metrics were 88.5% sensitivity (23/26) and 92.6% specificity (138/149), with an overall coincidence rate of 92.0% (161/175). Among the 175 field serum samples tested, 14 showed inconsistent results between cELISA and IFA. Of these, 11 were IFA-negative but cELISA-positive, which may be attributed to the higher analytical sensitivity of cELISA compared to IFA. The cELISA detected PDCoV-positive samples at a maximum dilution two-fold higher than that achieved by IFA. Conversely, the remaining 3 samples were IFA-positive but cELISA-negative. To further investigate these, the 3 discordant samples were analyzed using an N protein-based iELISA established in our lab, and 2 serum samples were positive. Given the documented serological cross-reactivity between PDCoV and PEDV, the 3 samples were also tested with a commercial PEDV IgA ELISA kit (Wuhan Keqian Biology Co., Ltd.), and all were positive. Although iELISA using the truncated PDCoV N protein (aa 217–342) can differentiate PDCoV-specific antibodies in clear background sera, its effectiveness in PDCoV-PEDV co-infection contexts remains unclear (Wang et al., 2025b). Furthermore, IFA detects antibodies against any PDCoV protein present in the infected cells, not solely the N protein. Collectively, potential cross-reactivity with PEDV and the presence of antibodies targeting viral proteins other than the N protein in some sera may account for the IFA-positive/cELISA-negative results.

In the present study, serum samples were collected from suckling piglets aged 7 to 21 days. It is reported that maternal IgG antibodies are transferred to piglets through colostrum within the first 24–48 h of life (Zhang et al., 2020). In a cELISA format, such maternal antibodies could potentially compete with the HRP-conjugated mAb for binding to the coated antigen, leading to false-positive results. As the maternal antibody status of the sampled piglets was not documented, the performance of the cELISA in the presence of maternal antibodies remains unknown.

## 5 Conclusion

In summary, we successfully generated a mAb (3C6) against the PDCoV N protein and established a cELISA. The developed cELISA proves to be a reliable and efficient serological tool for PDCoV detection.

## Data Availability

The original contributions presented in the study are included in the article/Supplementary material, further inquiries can be directed to the corresponding author.
