# Supplementary material for: Development of a nucleocapsid protein-based competitive ELISA for the detection of porcine deltacoronavirus antibodies
Source: Front Microbiol. 2025 Nov 26;16:1680835. doi: 10.3389/fmicb.2025.1680835 (PMC12689935; doi:10.3389/fmicb.2025.1680835)
Supplement: Supplementary file 1 [file Data_Sheet_1.docx]

**Supplementary figures**


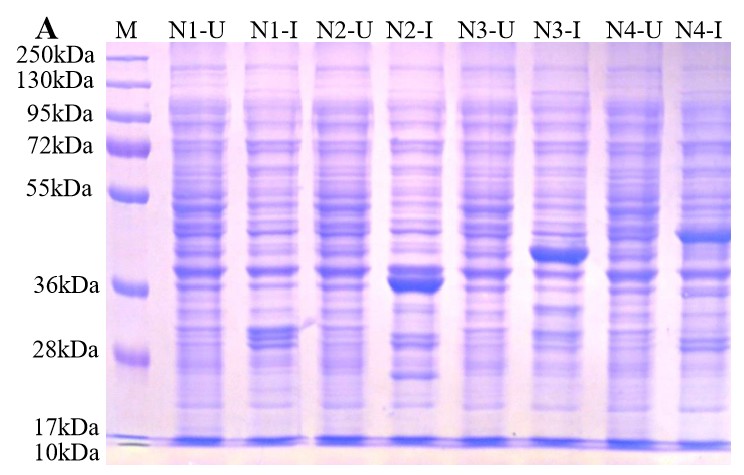


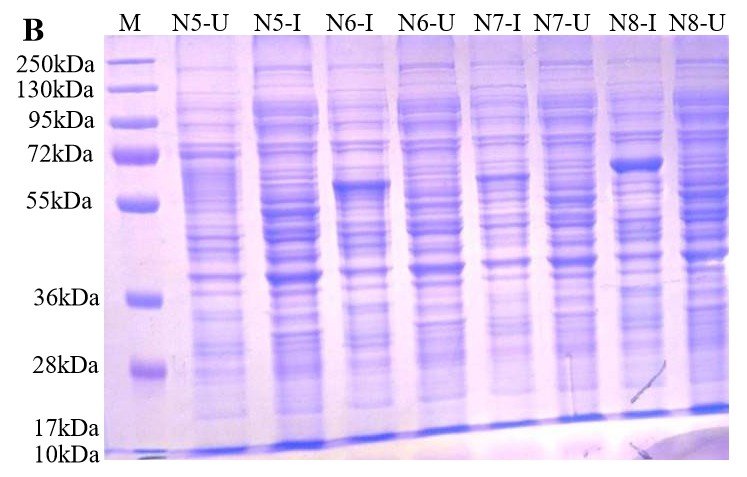


**Supplementary FIGURE S1. Expression of N1-N8 fragments of PDCoV N protein (aa 1-342) fused with GST tag in E.coli and analyzed using SDS-PAGE**.

Note: “U” denotes “uninduced” and “I” denotes “induced”. For instance, “N1-U” refers to cultures of E. coli BL21 (DE3) transformed with plasmid pGEX-4T-1-N1 that were not induced with IPTG, while “N1-I” refers to the same strain culture induced with IPTG.


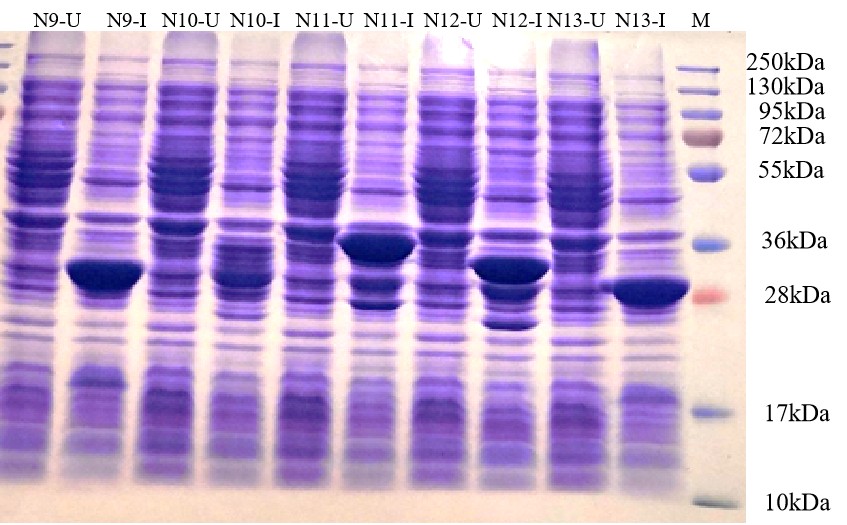


**Supplementary FIGURE S2. Expression of N9-N13 fragments of PDCoV N protein (aa 121-342) fused with GST tag in *E.coli* and analyzed using SDS-PAGE and Western blotting.** Note: “U” denotes “uninduced” and “I” denotes “induced”.


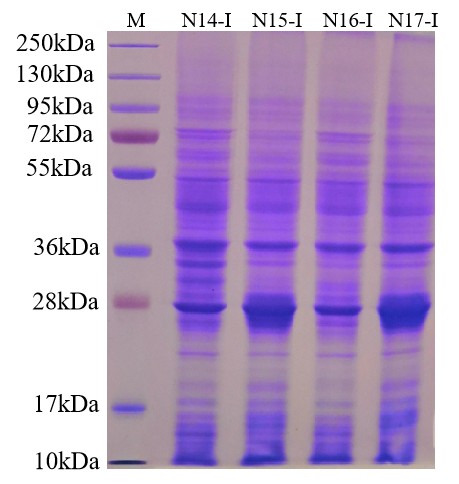


**Supplementary FIGURE S3. Expression of N14-N17 fragments of PDCoV N protein (aa 121-165) fused with GST tag in *E.coli* and analyzed using SDS-PAGE.**

Note: “I” denotes “induced”.

**Supplementary table**

Table Inter-rater agreement in the IFA results tested by the two blinded operators

|  | IFA | Operator A | | Total | Kappa | *p*-value |
| --- | --- | --- | --- | --- | --- | --- |
|  |  | Positive | Negative |  |  |  |
| Operator B | Positive | 146 | 3 | 149 | 0.889 | < 0.05 |
|  | Negative | 2 | 24 | 26 |  |  |
|  | Total | 148 | 27 | 175 |  |  |
